# Supplementary material for: Current Update on the Clinical Utility of MMSE and MoCA for Stroke Patients in Asia: A Systematic Review
Source: Int J Environ Res Public Health. 2021 Aug 25;18(17):8962. doi: 10.3390/ijerph18178962 (PMC8431226; doi:10.3390/ijerph18178962)
Supplement: Supplementary file 1 [file ijerph-18-08962-s001.zip › ijerph-1314084-supplementary.pdf]

Table S1. Terms used on database search.

Database	Search format
SCOPUS (N = 128)	(TITLE-ABS-KEY (*"cognitive assessment"* OR *tool* OR *screen* OR *test*) AND TITLE-ABS-KEY (*stroke*) AND TITLE-ABS-KEY (*cognitive* OR *cognition*) AND TITLE-ABS-KEY (*impairment* OR *deficit*) AND TITLE-ABS-KEY (*sensitivity* OR *specificity*) AND NOT TITLE-ABS-KEY (*animal* OR *review* OR *brain*)) AND (LIMIT-TO (DOCTYPE , *"ar"*)) AND (LIMIT-TO (EXACTKEYWORD , *"Human"*)) AND (LIMIT-TO (LANGUAGE , *"English"*))
Web of Science (N = 230)	**TOPIC:**((cognitive OR cognition)) *AND* **TOPIC:**((screening OR assessment OR tool OR test)) *AND* **TOPIC:** ((stroke OR "cerebrovascular accident")) *AND***TOPIC:** (("cognitive impair*" OR "cognitive deficit")) *AND* **TOPIC:**(sensitivity OR specificity) **Refined by: DOCUMENT TYPES:** (ARTICLE) **Timespan:** All years. **Indexes:** SCI-EXPANDED, SSCI, A&HCI, CPCI-S, CPCI-SSH, BKCI-S, BKCI-SSH, ESCI.
Science Direct (N = 162)	cognitive AND (test OR screen OR assessment OR tool) AND stroke AND sensitivity AND specificity AND impairment
PubMed (N = 786)	(assessment OR screen OR test OR tool) AND (cognitive OR cognition) AND (impairment OR deficit) AND (sensitivity OR specificity) AND (stroke OR CVA) NOT (review OR animal OR randomized OR motor)

Table S2. Detailed report on SE and SP for MMSE, MoCA, and NINDS-CNS 5.

Study	Cut-off value	SE, % (CI)	SP, % (CI)	PPV (CI)	NPV (CI)	AUC (CI)
Dong et al. 2012	MMSE ≤ 25/26	88	67	47	94	0.85 (0.80-0.91)
	MoCA ≤ 21/22	88	64	45	94	0.86 (0.81-0.91)
Tu et al. 2013	**NC vs. VCI-ND + VD**					
	MoCA ≤ 26/27	96	76	86	93	0.95 (0.93-0.97)
	**NC & VCI-ND vs. VD**					
	MoCA ≤ 16/17	93	96	91	97	0.99 (0.98-1.00)
Wu et al. 2013	**Not education-adjusted**					
	MoCA ≤ 22/23	65	79			0.80 (0.74-0.86)
	**Education ≤ 6 years**					
	MoCA ≤ 15	97	47			0.90 (0.83-0.98)
	**Education 6-12 years**					
	MoCA ≤ 22	56	88			0.81 (0.72-0.90)
	**Education > 12years**					
	MoCA ≤ 23	40	100			0.82 (0.68-0.96)
Dong et al. 2014	**NCI vs. VCI**					
	MMSE ≤ 26	71	82	84	67	0.84 (0.80-0.88)
	MoCA ≤ 23	78	80	84	72	0.87 (0.83-0.91)
	**NCI vs. Amnesic Global MCI**					
	MMSE ≤ 26	83	82	76	88	0.91 (0.86-0.94)
	MoCA ≤ 23	88	80	75	90	0.93 (0.89-0.96)
	**NCI vs. Non-amnestic Global MCI**					
	MMSE ≤ 27	80	64	19	97	0.83 (0.71-0.94)
	MoCA ≤ 22	87	88	45	98	0.94 (0.86-1.00)
	**NCI vs. Non-amnesic Single MCI**					
	MMSE ≤ 26	42	82	38	84	0.69
	MoCA ≤ 24	69	67	36	89	0.74
Chen et al. 2015	NINDS-CNS 5-Min ≤ 23.5	87 (72-95)	73 (57-85)	76 (60-87)	86 (69-95)	0.86 (0.78-0.94)
	NINDS-CNS 5-Min ≤ 24	92 (78-98)	68 (52-81)	73 (59-85)	90 (73-97)	
Dong et al. 2016	**Baseline**					
	MoCA ≤ 19/20	88	75	42	98	0.89
	NINDS-CSN 5-Min ≤ 7/8	73	75	41	92	0.8
	**3-6 months**					
	MoCA ≤ 20/21	83	80	50	95	0.9
	NINDS-CSN 5-min ≤ 7/8	70	83	49	92	0.83
Shen et al. 2016	**VCI-ND**					
	MMSE ≤ 27/28	82	78			0.84 (0.77-0.91)
	MoCA ≤ 23/24	86	75			0.88 (0.82-0.95)
Zuo et al. 2016	MoCA ≤ 22/23	85	88	91	80	0.85 (0.80-0.95)

Lim et al. 2017	NINDS-CSN 5-Min ≤ 6/7	82	67	33	95	0.74
Liao et al. 2020	MoCA ≤ 24	63	71	74	60	0.72
Zhu et al.	MMSE ≤ 27	68	82			0.81
2020	MoCA ≤ 21	64	90			0.82

Notes: MMSE = Mini Mental Status Examination, MoCA = Montreal Cognitive Assessment, NINDS-CNS 5-Min = National Institute of Neurological Disorders and Stroke-Canadian Stroke Network 5-Minute Protocol, NIHSS = National Institute of Health Stroke Severity Scale; CDR = Clinical Dementia Rating, SVID = Subcortical Vascular Ischemic Dementia, VCI = Vascular cognitive impairment; PPV = Positive predictive value, NPV = Negative predictive value, AU = Area under curve, CI = 95% Confidence interval.
